# The Efficacy of Natalizumab versus Fingolimod for Patients with Relapsing-Remitting Multiple Sclerosis: A Systematic Review, Indirect Evidence from Randomized Placebo-Controlled Trials and Meta-Analysis of Observational Head-to-Head Trials

**DOI:** 10.1371/journal.pone.0163296

**Published:** 2016-09-29

**Authors:** Georgios Tsivgoulis, Aristeidis H. Katsanos, Dimitris Mavridis, Nikolaos Grigoriadis, Efthymios Dardiotis, Ioannis Heliopoulos, Panagiotis Papathanasopoulos, Theodoros Karapanayiotides, Constantinos Kilidireas, Georgios M. Hadjigeorgiou, Konstantinos Voumvourakis, HELANI (Hellenic Academy of Neuroimmunology)

**Affiliations:** 1 Second Department of Neurology, “Attikon” Hospital, School of Medicine, University of Athens, Athens, Greece; 2 Department of Neurology, The University of Tennessee Health Science Center, Memphis, Tennessee, United States of America; 3 International Clinical Research Center, Department of Neurology, St. Anne’s University Hospital in Brno, Brno, Czech Republic; 4 Department of Neurology, School of Medicine, University of Ioannina, Ioannina, Greece; 5 Department of Primary Education, University of Ioannina, Ioannina, Greece; 6 Department of Hygiene and Epidemiology, School of Medicine, University of Ioannina, Ioannina, Greece; 7 Second Department of Neurology, “AHEPA” University Hospital, Aristotelion University of Thessaloniki, Thessaloniki, Macedonia, Greece; 8 Department of Neurology, University Hospital of Larissa, University of Thessaly, Larissa, Greece; 9 Department of Neurology, Alexandroupolis University Hospital, Democritus University of Thrace, Alexandroupolis, Greece; 10 Department of Neurology, University of Patras Medical School, Patras, Greece; 11 First Department of Neurology, “Eginition” Hospital, School of Medicine, University of Athens, Athens, Greece; University of Oxford, UNITED KINGDOM

## Abstract

**Background:**

Although Fingolimod (FGD) and Natalizumab (NTZ) appear to be effective in relapsing-remitting multiple sclerosis (RRMS), they have never been directly compared in a randomized clinical trial (RCT).

**Methods and Findings:**

We evaluated the comparative efficacy of FGD vs. NTZ using a meta-analytical approach. Data from placebo-controlled RCTs was used for indirect comparisons and observational data was utilized for head-to-head comparisons. We identified 3 RCTs (2498 patients) and 5 observational studies (2576 patients). NTZ was associated with a greater reduction in the 2-year annualized relapse rate (ARR; SMD_indirect_ = -0.24;95% CI: from -0.44 to -0.04; p = 0.005) and with the probability of no disease activity at 2 years (OR_indirect_:1.82, 95% CI: from 1.05 to 3.15) compared to FGD, while no differences between the two therapies were found in the proportion of patients who remained relapse-free (OR_indirect_ = 1.20;95% CI: from 0.84 to 1.71) and those with disability progression (OR_indirect_ = 0.76;95% CI: from 0.48 to 1.21) at 2 years. In the analysis of observational data, we found no significant differences between NTZ and FGD in the 2-year ARR (SMD = -0.05; 95% CI: from -0.26 to 0.16), and 2-year disability progression (OR:1.08;95% CI: from 0.77 to 1.52). However, NTZ-treated patients were more likely to remain relapse-free at 2-years compared to FGD (OR: 2.19;95% CI: from 1.15 to 4.18; p = z0.020).

**Conclusions:**

Indirect analyses of RCT data and head-to-head comparisons of observational findings indicate that NTZ may be more effective than FGD in terms of disease activity reduction in patients with RRMS. However, head-to-head RCTs are required to independently confirm this preliminary observation.

## Introduction

Available disease modifying drugs (DMDs) have been proved to be effective in reducing disability progression in patients with relapsing remitting multiple sclerosis (RRMS) [1]. High-risk RRMS patients with active or progressive disease while on treatment with a first-line agent are candidates for treatment escalation to a second-line agent, which is expected to have a more potent effect on both clinical and MRI outcomes [2]. Second-line therapy should thus be chosen after careful risk-benefit ratio stratification [3,4]. Although the second-line agents Fingolimod and Natalizumab appear to be efficacious for patients with high disease activity and generally manageable side effects [2], their comparative efficacy in patients with RRMS has never been tested within the setting of a randomized clinical trial (RCT).

The aim of the present systematic review and meta-analysis is to compare the relative efficacy of Natalizumab and Fingolimod in RRMS patients by estimating an indirect effect using available randomized placebo-control trials and by estimating an effect from observational studies on the reported efficacy outcomes.

## Methods

### Trial identification and data abstraction

This meta-analysis is presented according to the Preferred Reporting Items for Systematic Reviews and Meta-Analyses (PRISMA) guidelines for systematic reviews and meta-analyses [5]. We performed a comprehensive literature search in MEDLINE, SCOPUS and the CENTRAL Register of Controlled Trials databases to identify: 1. all eligible placebo-control RCTs of Natalizumab or Fingolimod in RRMS patients and 2. all eligible observational studies comparing Natalizumab to Fingolimod in RRMS patients. The following keywords were used in all database searches: “relapsing-remitting multiple sclerosis”, “RRMS”, “fingolimod” and “natalizumab”. We imposed no language or other restrictions. Last literature search was performed on April 16th, 2016. We examined reference lists of all retrieved articles to identify studies that may have been missed by the initial database search. References to studies were also sourced from international trials registers via the World Health Organization’s (WHO) trials portal *(http://apps.who.int/trialsearch/)*; regulatory agencies; drug companies; the hand-searching of key journals, conference proceedings and other (non-Cochrane) systematic reviews and meta-analyses.

Database search was performed independently by three reviewers (GT, AHK, KV), while all emerging disagreements were resolved with consensus. We excluded from further quantitative/qualitative analysis all: 1. case series/ case reports, 2.RCTs without placebo arms, 3.studies reporting combination therapy in the treatment arm, 4. studies not reporting the outcomes of interest and 5. Phase II core study protocols with inadequate follow-up time (<1 year), since in multiple sclerosis the clinically relevant endpoint, which documents the presence or absence of progressive disability, consists of structured observations with validated imaging assessment over a long period of time (usually 2 years) [6], while the differences in the annualized relapse rate (ARR) are considered to be evident after an observational period of at least 1 year [7].

Data on the ARR, percentage of patients with disability progression, percentage of patients who were free of relapses and percentage of patients with no evidence of disability progression (NEDA) during the study period were extracted independently by the same authors who performed the literature search (GT, AHK, KV) for all arms in both randomized and observational study protocols that were included for the quantitative synthesis. NEDA was defined as the occurrence of no relapses, no progression of disability sustained for 12 weeks, no gadolinium-enhanced lesions and no new or enlarging T2-hyperintense lesions on MRI scan [8].

We calculated odds ratios (ORs) to express the comparison of the reported dichotomous outcomes for each available subgroup in each study protocol. The equivalent z-test was performed for each OR, and if p < 0.05 it was considered statistically significant. We expressed the unadjusted mean differences of reported continuous outcomes between subgroups as standardized mean differences (SMDs). SMD estimates were calculated as the mean differences divided by the corresponding pooled standard deviations and were subsequently interpreted using a general rule of thumb reported by Cohen, in which an SMD of 0.2 represents a small effect, an SMD of 0.5 represents a medium effect, and an SMD of 0.8 or larger represents a large effect [9,10]. To make the interpretation of SMDs more clinically relevant we additionally re-expressed all SMDs as ORs using the formula OR = exp[(SMDxπ)/sqrt(3)] and after assuming that the underlying continuous measurements in each group follow a logistic distribution and that the variability of the outcomes is the same in both FGD and NTZ group [11].

We performed meta-analysis for all the aforementioned outcomes of the included RCTs that reported treatment arms with any of the two drugs (Natalizumab or Fingolimod) versus the corresponding placebo arms, and meta-analysis for the same outcomes among patients receiving Natalizumab versus those receiving Fingolimod in the included observational studies. For the included RCTs we performed subsequent subgroup analyses, dichotomizing studies according to the reported treatment arm (Natalizumab or Fingolimod) in each study protocol. The mixed-effects model was used to calculate both the pooled point estimate in each subgroup and the overall estimates in all occasions. According to the mixed-effects model, we used a random effects model (DerSimonian Laird) to combine studies within each subgroup and a fixed effect model (Mantel–Haenszel method) to combine subgroups and estimate the overall effect. We assumed the between-study variance (tau-squared) to be the same for all subgroups. Tau-squared was first computed within subgroups and then pooled across subgroups [12]. We assessed heterogeneity between studies with the Cochran Q and I^2^ statistics. For the qualitative interpretation of heterogeneity, I^2^ values of at least 50% were considered to represent substantial heterogeneity, while values of at least 75% indicated considerable heterogeneity, as per the Cochrane Handbook [13].

In RCTs we compared the pooled effect sizes (SMDs or ORs) for each outcome of interest between Natalizumab and Fingolimod treatment arms by calculating the indirect effect sizes (indirect SMDs and indirect ORs), with their corresponding 95% confidence intervals, using the Bucher’s Method [14]. As transitivity assumption is a key assumption for the indirect effect to be valid, we compared the baseline characteristics of RRMS patients included in the corresponding RCTs that were treated with natalizumab and those treated with fingolimod to explore if effect modifiers are similarly distributed across the two comparisons [15].

Statistical analyses were conducted using the Review Manager (RevMan) Version 5.3 software (Copenhagen: The Nordic Cochrane Centre, The Cochrane Collaboration, 2014) and the Stata Statistical Software (StataCorp. 2013. Stata Statistical Software: Release 13. College Station, TX: StataCorp LP).

## Results

### Study selection and study characteristics

**Fig 1:**
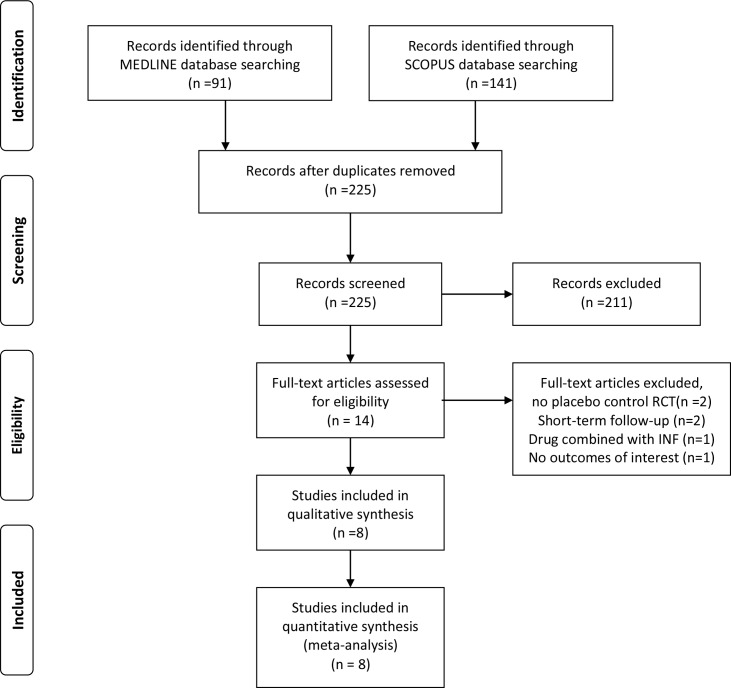
Flow chart presenting the selection of eligible studies.

Systematic search of MEDLINE and SCOPUS databases yielded 91 and 141 results respectively. Subsequent search in the CENTRAL Register of Controlled Trials retrieved no additional RCTs. After removing duplicates, the titles and abstracts from the remaining 225 studies were screened and 14 potentially eligible studies for the meta-analysis were retained. The complete search algorithm used in the MEDLINE search is available in the S1 File. After retrieving the full-text version of the aforementioned 14 studies, 6 studies were excluded because they either reported non-placebo control RCTs or combination therapy with interferon (INF) or short-term follow-up (6 months) or not providing data on the outcomes of interest. All excluded studies with reasons for exclusion are presented in Table A in S1 File. In the final presentation of the literature search results, there was no conflict or disagreement between the 3 reviewers and the 8 studies that met the study protocol’s inclusion criteria were included in both the qualitative and quantitative synthesis (Fig 1).

The baseline characteristics of the arms of RRMS patients that were included in available placebo-control RCTs (2498 total patients) and treated with either Natalizumab or Fingolimod are presented in Table 1 [16–18]. After comparing available variables, using t-test and chi-square statistical tests where appropriate, we found that patients randomized to receive Natalizumab in the AFFIRM trial [16] were significantly younger but had a higher pre-treatment load of gadolinium-enhancing lesions compared to the patients randomized to receive Fingolimod in the FREEDOMS I&II trials [17,18].

**Table 1:** Baseline characteristics of patients in the included Randomized Clinical Trials.

	Natalizumab	Fingolimod	p-value
RCTs	AFFIRM [16]	FREEDOMS I [17], FREEDOMS II [18]	
Patients (n)	627	783	
Age (years±SD)	35.6±8.5	38.5±8.6	<0.001
Males (n, %)	178 (28%)	212 (27%)	0.675
Disease duration (median, years)	5.0	N/A	N/A
History of previous DMT	N/A*	43.2%	-
Relapses in previous year (mean±SD)	1.53±0.91	1.46±0.84	0.134
Baseline EDSS (mean±SD)	2.3±1.2	2.3±1.3	1.0
Gd+ lesions (mean±SD)	2.2±4.7	1.4±4.2	<0.001
≥9 T2-MRI lesions	597 (95%)	N/A	N/A

n: number, SD: standard deviation, DMT: disease modifying treatment, Gd+: gadolinium enhancing, N/A: not available

*patients receiving treatment with cyclophosphamide or mitoxantrone within the previous year, or treatment with interferon beta, glatiramer acetate, cyclosporine, azathioprine, methotrexate, or intravenous immune globulin within the previous 6 months or treatment with interferon beta, glatiramer acetate, or both for more than six months were excluded.

Similarly, the baseline characteristics of RRMS patients treated with either Natalizumab of Fingolimod in all eligible prospective observational studies (2576 total patients) are presented in Table B in S1 File [19–23]. Among studies patients receiving Natalizumab were reported to be younger than patients receiving Fingolimod [20], while patients receiving Natalizumab were reported to have more severe pre-treatment EDSS score [19–22], higher pre-treatment load of gadolinium-enhancing lesions [19] and more relapses before treatment [19–22]. Four of the study protocols reported statistical methods for either balancing the baseline characteristics between subgroups or addressing for potential confounders [19–22], while one of the studies reported only unadjusted estimates between subgroups (Table 2) [23].

**Table 2:** Significant differences among patients with relapsing-remitting multiple sclerosis treated with natalizumab and patients with relapsing-remitting multiple sclerosis treated with fingolimod in the included observational study protocols and reported methods for confounders adjustment.

Authors, year	Significant differences in baseline characteristics among subgroups	Method for confounders adjustment
Barbin et al, 2016 [19]	NTZ treated patients had higher mean EDSS, higher number of relapses & higher percentage Gd+ lesions	inverse probability treatment weighting
Braune et al, 2013 [20]	FGD treated patients had higher mean age/ NTZ treated patients had higher mean EDSS & higher number of relapses	N/R
Gajofatto et al, 2014 [21]	NTZ treated patients had higher EDSS score and higher number of relapses	Multivariate Cox and logistic regression models
Kalincik et al, 2015 [22]	N/R	Propensity score matching
Koch-Henriksen et al, 2015 [23]	None	Propensity score matching

NTZ: natalizumab, FGD: fingolimod, N/R: not reported

### Overall analysis and indirect estimates in randomized clinical trials

**Fig 2:**
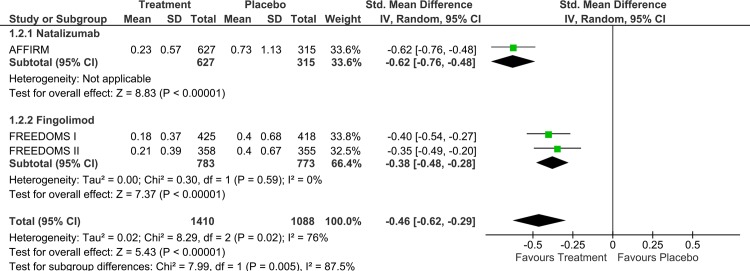
Analysis on the annualized relapse rate reduction at 2 years reported for patients with relapsing remitting multiple sclerosis included in the randomized clinical trials of Natalizumab or Fingolimod.

Natalizumab was found to be associated with a greater reduction in the 2-year ARR compared to placebo (SMD: -0.62; 95% CI: from -0.76 to -0.48; Fig 2 and OR:0.32; 95%CI: from 0.25 to 0.41; Fig A in S1 File) than the ARR reduction of Fingolimod in 2 years compared to placebo (SMD: -0.38, 95% CI: from -0.48 to -0.28; Fig 2 and OR:0.50 95%CI: from 0.42 to 0.60; Fig A in S1 File). The p-value for subgroup differences was 0.005 (SMD_indirect_: -0.24; 95% CI: from -0.44 to -0.04 and OR_indirect_: 0.64; 95%CI: from 0.45 to 0.93). No evidence of heterogeneity was found in both subgroups (I^2^ = 0).

However, the percentage of patients with no relapse at 2 years was not found to be significantly different among the RRMS patients treated with Natalizumab and those treated with Fingolimod (OR for Natalizumab: 3.04, 95% CI: from 2.29 to 4.03 vs OR for Fingolimod: 2.54, 95% CI: from 2.05 to 3.17, p-value for subgroup differences:0.33, Fig B in S1 File; OR_indirect_:1.20, 95% CI: from 0.84 to 1.71). No evidence of heterogeneity was present (I^2^ = 3%, p-value for chi-square test statistic Q: 0.36). Similarly, the percentage of patients with disability progression at 2 years did not differ between RRMS patients treated with Natalizumab and Fingolimod (OR for Natalizumab: 0.51, 95% CI: from 0.37 to 0.70 vs OR for Fingolimod: 0.67, 95% CI: from 0.48 to 0.94, p-value for subgroup differences: 0.23, Fig C in S1 File; OR_indirect_: 0.76, 95% CI: from 0.48 to 1.21). No evidence of heterogeneity was present (I^2^ = 30%, p-value for chi-square test statistic Q: 0.23).

**Fig 3:**
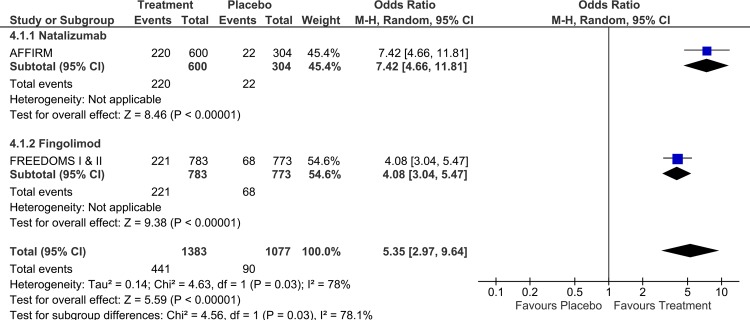
Analysis on the proportion of patients with no evidence of disease activity at 2-years included in the randomized clinical trials of Natalizumab or Fingolimod.

Finally, a significantly higher percentage of RRMS with NEDA at 2-years was found in patients randomized to receive Natalizumab than those randomized to receive Fingolimod in the corresponding RCTs [8,23] (OR for Natalizumab: 7.42, 95%CI: from 4.66 to 11.81 vs OR for Fingolimod: 4.08, 95%CI: from 3.04 to 5.47, p-value for subgroup differences:0.03, Fig 3; OR_indirect_:1.82, 95% CI: from 1.05 to 3.15).

### Overall and subgroup analyses in observational study data

In the subsequent analysis of all available observational study data no significant difference (p = 0.66) in the 2-year ARR was found among Natalizumab and Fingolimod (SMD:-0.05, 95% CI: from -0.26 to 0.16; Fig D in S1 File and OR: 0.92; 95%CI: from 0.64 to 1.34; Fig E in S1 File). However, substantial heterogeneity was present within studies (I^2^ = 64%, p-value for chi-square test statistic Q: 0.06). Similarly, no significant difference in the proportion of patients with disability progression was observed between RRMS patients treated with Natalizumab and those treated with Fingolimod at both 1-year (OR: 1.37, 95% CI: from 0.95 to 1.98, p-value = 0.10) and 2-years (OR: 1.08, 95% CI: from 0.77 to 1.52; p-value = 0.36; Fig F in S1 File), with no evidence of heterogeneity among estimates (I^2^<40% for both subgroups). Finally, in another subgroup analysis patients treated with Natalizumab were found to have a significantly higher proportion of relapse-free patients at 2-years patients compared to those treated with Fingolimod (OR: 2.19, 95% CI: from 1.15 to 4.18, p-value = 0.02; Fig G in S1 File). However, this difference was marginally not significant during the first year (OR: 1.61, 95% CI: from 0.94 to 2.78, p-value = 0.09; Fig G in S1 File) and considerable heterogeneity was observed within studies for both the 1^st^ and 2^nd^ year (I^2^>80%).

## Discussion

**Fig 4:**
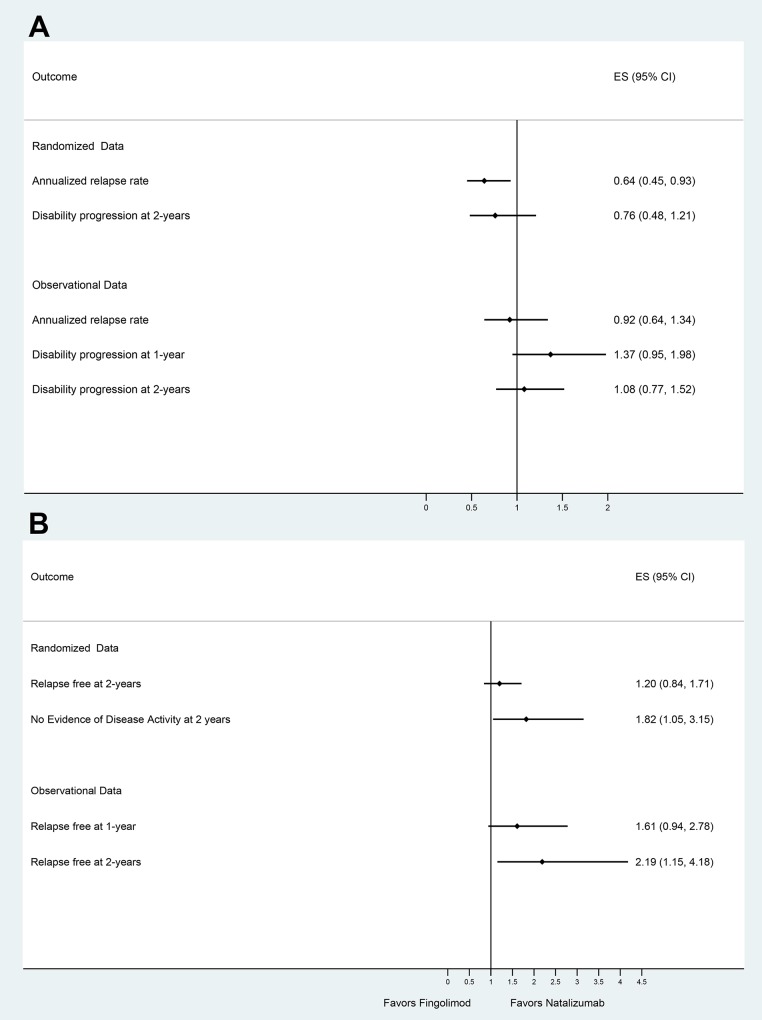
Indirect estimates from randomized clinical trials and estimates from observational studies with their corresponding 95% confidence intervals for (A) positive effect sizes and (B) negative effect sizes of the outcomes of interest reported as odds ratios between patients with relapsing remitting multiple sclerosis receiving natalizumab and those receiving fingolimod.

Both the indirect analyses of available randomized data and the direct comparisons from observational data indicate that RRMS patients receiving Natalizumab have a greater reduction in the 2-year ARR and are more likely to remain relapse-free and to achieve NEDA-3 status at two years [24], when compared to those treated with Fingolimod (Fig 4).

Even though no significant differences between the two therapies were found in terms of disability progression, we should take into consideration that in baseline characteristic analyses of both RCT and observational data, patients receiving Natalizumab had a more aggressive disease profile at baseline compared to those receiving Fingolimod (higher pre-treatment EDSS score, higher pre-treatment load of gadolinium-enhancing lesions and more relapses before treatment initiation). Moreover, AFFIRM trial [16] excluded patients who were treated with DMDs for more than six months, while previous treatment with immunomodulatory agents was highly prevalent in FREEDOMS (42.6%) [17] and FREEDOMS II (74%) [18]. Consequently, it may be postulated that patients randomized to natalizumab had more active disease (due to lack of long-term pretreatment) in comparison to patients randomized to fingolimod who were treatment-naïve in 43.2% (Table 1). Apart from the aforementioned discrepancies in baseline characteristics between treatments, the lack of significant differences in disability progression could also be partially explained by the continuously declining trend of EDSS progression over time that has been observed in RCTs [25], which inevitably may lead to underpowered estimates and thus only subtle differences among subgroups.

Results of a 9-year multicenter, cross-sectional, clinical-MRI study of 241 patients with RRMS suggest that baseline EDSS and baseline MRI are the best long-term predictors of disease progression [26]. The increase in EDSS score from baseline to 2-years in RRMS patients under treatment with IFN β-1a was found to be an independent predictor of both EDSS progression and conversion to the secondary progressive form of the disease over a 15 year period [27]. A post-hoc individual patient data analysis from a large, placebo-controlled trial of INFβ-1a in RRMS also highlighted the predictive role of emerging MRI lesions and relapses during the first year on the 2-year EDSS progression [28]. More specifically, baseline lesion number on T1 and T2 sequences and the number of new or enlarging T2 lesions were found to be independent predictors of sustained disability progression over a 12-year observation period [29]. Gadolinium-enhancing (Gd+) lesions are also considered to be surrogate markers of treatment effect on relapse rate and disability progression in RRMS patients [30], as gadolinium enhancement on MRI represents blood-brain barrier instability and thus ongoing disease activity [31]. Thus both the presence of multiple Gd+ lesions at baseline MRI evaluation and the occurrence of new lesions during the first several years of the disease have been associated with worse long-term prognosis [32, 33].

Apart from radiological findings, the number of relapses during the disease course has also been highlighted to be an independent and significant predictor of disease progression [34]. Consequently, it may be postulated that the reduced ARR that was documented in patients treated with Natalizumab (in comparison to Fingolimod treatment) may also translate into reduced rate of disability progression in a larger sample of RRMS with a longer duration of follow-up. The higher rate of NEDA-3 status in patients randomized to Natalizumab in comparison to Fingolimod that was documented in the indirect comparison of RCT data lends support to the former hypothesis. In a longitudinal cohort of patients with an initial diagnosis of RRMS or clinically isolated syndrome, NEDA-3 status at 2 years was found to be a significant predictor of disease progression throughout a 7-year follow-up period [35]. These data suggest that NEDA status is of paramount importance to detect disease activity early in the course of the disease, and thus influence disease progression with a timely and optimal treatment selection [36]. To the best of our knowledge, this is the first-meta-analysis on the comparative efficacy of different DMDs that has incorporated NEDA-III status as an endpoint.

Apart from the efficacy endpoints, a comparative analysis of first-year Fingolimod and Natalizumab drug discontinuation among Swedish RRMS patients suggests that both drugs are well tolerated therapies, but fingolimod is less tolerated in the subgroup of patients switching from Natalizumab [37]. In a cost-effective analysis based on the perspective of the Swedish healthcare system Fingolimod treatment was found to be less expensive, but treatment with Natalizumab was found to be more effective, resulting in a relapse decrease and thus a better cost-to-benefit ratio, especially for patients with rapidly evolving disease [38]. The cost-effectiveness of Natalizumab in patients with highly active RRMS has also been confirmed in another cost-effective analysis based on the UK healthcare system [39].

Our results are in agreement with a very recent network meta-analysis on RCT data from immunomodulators and immunosuppressants that have been used for the treatement of RRMS, in which Natalizumab was ranked as the third more effective treatment [RR 0.56, 95% CI 0.47 to 0.66; surface under the cumulative area curve (SUCRA) 88%; high quality evidence] used in RRMS, followed by Fingolimod (RR 0.72, 95% CI 0.64 to 0.81; SUCRA 71%; moderate quality evidence) [40]. The authors also noted that Natalizumab was the only therapy to provide moderate quality evidence in the prevention of the 2-year disability progression, with all other treatments providing low to very low quality of evidence. Even though the authors of the aforementioned network meta-analysis [40] reported that they found no evidence of important variables variations across comparisons, we found significant differences in baseline characteristics between patients randomized to receive Natalizumab and those randomized to receive Fingolimod (Table 1). We also highlighted that the imbalances found in baseline characteristics from RCTs were also present in the observational studies, suggesting that all indirect analyses between Natalizumab and Fingolimod should be interpreted with caution and awareness of the population imbalances in baseline disease severity.

Several limitations should be acknowledged for the correct interpretation of the present report. First, even though indirect meta-analysis is very helpful in comparing the relative effectiveness and acceptability of competing treatments, several issues need to be appropriately addressed for the results to be valid and correctly interpreted [41]. Transitivity assumption, which implies that the distribution of potential effect modifiers is the same across treatment comparisons, is a key element that should be present before the conduction of all indirect analyses [41]. Although comparison of the subgroups in the included RCTs revealed imbalances in baseline characteristics between patients randomized to treatment with Natalizumab and those treated with Fingolimod (Table 1), we decided to perform indirect analyses but also take into consideration that the indirect outcomes could likely provide an underestimated effect of Natalizumab compared to Fingolimod due to the higher baseline disease severity that was present in patients randomized to Natalizumab compared to Fingolimod in the corresponding RCTs. Moreover, only 3 placebo-control RCTs (1 with Natalizumab [16] & 2 with Fingolimod [17,18]) were available for inclusion in the present analysis and thus we were unable to reliably investigate the presence of heterogeneity between RCTs. In most of the observational study analyses, evidence of considerable or substantial heterogeneity was present. Three of the observational study protocols reported significant imbalances in patients’ baseline characteristics [19–21], one study reported no significant differences among the subgroups of patients treated with Natalizumab and those treated with Fingolimod [23], while the remaining study provided no report on potential differences between baseline characteristics [22]. However, it should be noted that all observational study protocols, except for one [20], provided adjusted outcome measures after adjusting for potential confounders. Finally, apart from the baseline disease severity and MRI burden, it should be noted that John Cunningham virus (JCV) antibody serologic testing and the corresponding risk of progressive multifocal leukoencephalopathy (PML) have a significant role in the final decision to use Natalizumab versus Fingolimod as a second-line treatment for patients with RRMS [42, 43].

In conclusion, available randomized and observational study data suggest that Natalizumab is probably more effective than Fingolimod in terms of relapse reduction and NEDA-III status increase in patients with RRMS. Head-to-head RCTs that will directly compare the two aforementioned therapeutic options are required to independently confirm this preliminary observation.

## Supporting Information

S1 File MEDLINE Search algorithm. **Table A.** Excluded studies with reasons for exclusion. **Table B.** Baseline characteristics of included observation studies. **Fig A.** Analysis on the annualized relapse rate odds ratios of patients with relapsing remitting multiple sclerosis included in the randomized clinical trials of Natalizumab or Fingolimod. **Fig B.** Analysis on the proportion of patients with relapsing remitting multiple sclerosis included in the randomized clinical trials of Natalizumab or Fingolimod who had absence of relapse reduction at 2 years. **Fig C.** Analysis on the proportion of patients with relapsing remitting multiple sclerosis included in the randomized clinical trials of Natalizumab or Fingolimod who had disability progression at 2 years. **Fig D.** Subgroup analysis on the annualized relapse rate reduction in observational studies of patients with relapsing remitting multiple sclerosis receiving treatment with Natalizumab of Fingolimod. **Fig E.** Subgroup analysis on the annualized relapse rate odds ratios in observational studies of patients with relapsing remitting multiple sclerosis receiving treatment with Natalizumab of Fingolimod. **Fig F.** Subgroup analysis on the proportion of relapsing remitting multiple sclerosis patients with disability progression receiving treatment with Natalizumab of Fingolimod in observational study protocols at both the first and second year. **Fig G.** Subgroup analysis on the proportion of relapsing remitting multiple sclerosis patients with no relapses receiving treatment with Natalizumab of Fingolimod in observational study protocols at both the first and second year.
(DOC)

S2 File PRISMA checklist. (DOCX)
