# Supplementary material for: The Efficacy of Natalizumab versus Fingolimod for Patients with Relapsing-Remitting Multiple Sclerosis: A Systematic Review, Indirect Evidence from Randomized Placebo-Controlled Trials and Meta-Analysis of Observational Head-to-Head Trials
Source: PLoS One. 2016 Sep 29;11(9):e0163296. doi: 10.1371/journal.pone.0163296 (PMC5042498; doi:10.1371/journal.pone.0163296)
Supplement: S1 File — Table A. Excluded studies with reasons for exclusion. Table B. Baseline characteristics of included observation studies. Fig A. Analysis on the annualized relapse rate odds ratios of patients with relapsing remitting multiple sclerosis included in the randomized clinical trials of Natalizumab or Fingolimod. Fig B. Analysis on the proportion of patients with relapsing remitting multiple sclerosis included in the randomized clinical trials of Natalizumab or Fingolimod who had absence of relapse reduction at 2 years. Fig C. Analysis on the proportion of patients with relapsing remitting multiple sclerosis included in the randomized clinical trials of Natalizumab or Fingolimod who had disability progression at 2 years. Fig D. Subgroup analysis on the annualized relapse rate reduction in observational studies of patients with relapsing remitting multiple sclerosis receiving treatment with Natalizumab of Fingolimod. Fig E. Subgroup analysis on the annualized relapse rate odds ratios in observational studies of patients with relapsing remitting multiple sclerosis receiving treatment with Natalizumab of Fingolimod. Fig F. Subgroup analysis on the proportion of relapsing remitting multiple sclerosis patients with disability progression receiving treatment with Natalizumab of Fingolimod in observational study protocols at both the first and second year. Fig G. Subgroup analysis on the proportion of relapsing remitting multiple sclerosis patients with no relapses receiving treatment with Natalizumab of Fingolimod in observational study protocols at both the first and second year. (DOC) [file pone.0163296.s001.doc]

**S1 File**

**Complete search algorithm used in Medline search**

(("fingolimod hydrochloride"[MeSH Terms] OR ("fingolimod"[All Fields] AND "hydrochloride"[All Fields]) OR "fingolimod hydrochloride"[All Fields] OR "fingolimod"[All Fields]) OR ("natalizumab"[MeSH Terms] OR "natalizumab"[All Fields])) AND ("multiple sclerosis, relapsing-remitting"[MeSH Terms] OR ("multiple"[All Fields] AND "sclerosis"[All Fields] AND "relapsing-remitting"[All Fields]) OR "relapsing-remitting multiple sclerosis"[All Fields] OR ("relapsing"[All Fields] AND "remitting"[All Fields] AND "multiple"[All Fields] AND "sclerosis"[All Fields]) OR "relapsing remitting multiple sclerosis"[All Fields]) AND Clinical Trial[ptyp]

**SUPPLEMENTAL TABLES**

**Table A.** Excluded studies with reasons for exclusion

| **Authors, year** | **Reason(s) for exclusion** |
| --- | --- |
| Carruthers et al, 2014 [1] | No outcome measures provided |
| Cohen et al, 2010 [2] | No placebo subgroup (Interferon comparator) |
| Kappos et al, 2006 [3] | 6-month follow-up (Core study) |
| Kappos et al, 2015 [4] | No placebo subgroup (Extension study) |
| Miller et al, 2003 [5] | 6-month follow-up (Core study) |
| Rudick et al, 2006 [6] | Natalizumab combined with Interferon |

**Table B.** Baseline characteristics of included observation studies

| **Authors, year** | **Country** | **Total patients (NTZ/FGD)** | **Age, mean ±SD (NTZ/FGD)** | **Male gender, % (NTZ/FGD)** | **Baseline EDSS, mean ±SD or median (range) (NTZ/FGD)** | **Years of disease duration, mean±SD or median (range) (NTZ/FGD)** | **No Gd+ lesions on baseline MRI, % (NTZ/FGD)** |
| --- | --- | --- | --- | --- | --- | --- | --- |
| Barbin et al, 2016 [7] | France | 629  (326/ 303) | 36.8±9.9/ 37.2±9.2 | 25.7%/ 22.1% | 2.8±1.3/ 2.4±1.3* | 8.0±.6.1/ 9.0±6.8 | 56.8/ 41.6* |
| Braune et al, 2013 [8] | Germany | 427  (237/ 190) | 37.4±9.6/ 40.5±8.7* | 30.4%/ 32.1% | 3.3±1.8/ 2.3±1.6* | 9.1±6.9/ 9.9±6.9 | NR/NR |
| Gajofatto et al, 2014 [9] | Italy | 87 (57/ 30) | 38.0±9.3/ 39.0±7.8 | 24.6%/ 30.0% | 3 (2-8)/ 2.5(0-5.5)* | 8.4 (0.5-31.5) | 40.4/ 26.7 |
| Kalincik et al, 2015 [10] | International | 578  (407/ 171) | 37.0±9.0/ 38.0±10.0 | 26.0%/ 26.0% | 3.4±1.5/ 3.1±1.7 | 9.4±6.2/ 9.5±8.0 | 15/ 9 |
| Koch-Henriksen et al, 2015 [11] | Denmark | 942  (464/ 464) | 38.7±10.1/ 39.3±10.1 | 29.5%/ 29.5%/ | 3.15±1.6/ 3.08±1.05 | 7.8±6.2/ 7.7±6.3 | NR/NR |

NTZ: natalizumab, FGD: fingolimod, n: number, SD: standard deviation, EDSS: expanded disability status scale, Gd+: gadolinium enhancing, N/R: not reported, *statistically significant differences (p<0.005)

**Fig A.** Analysis on the annualized relapse rate odds ratios of patients with relapsing remitting multiple sclerosis included in the randomized clinical trials of Natalizumab or Fingolimod.


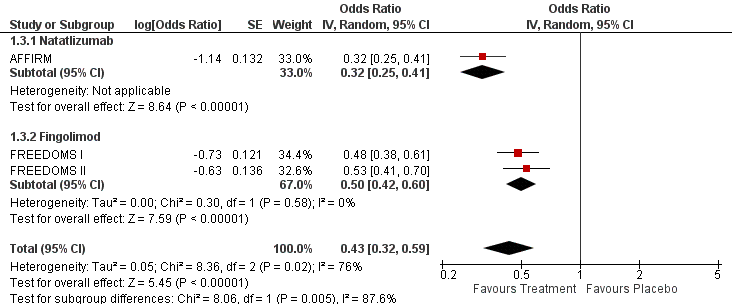


**Fig B.** Analysis on the proportion of patients with relapsing remitting multiple sclerosis included in the randomized clinical trials of Natalizumab or Fingolimod who had absence of relapse reduction at 2 years.


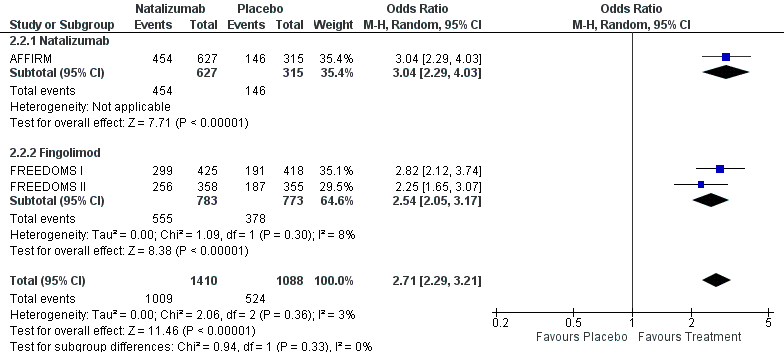


**Fig C.** Analysis on the proportion of patients with relapsing remitting multiple sclerosis included in the randomized clinical trials of Natalizumab or Fingolimod who had disability progression at 2 years.


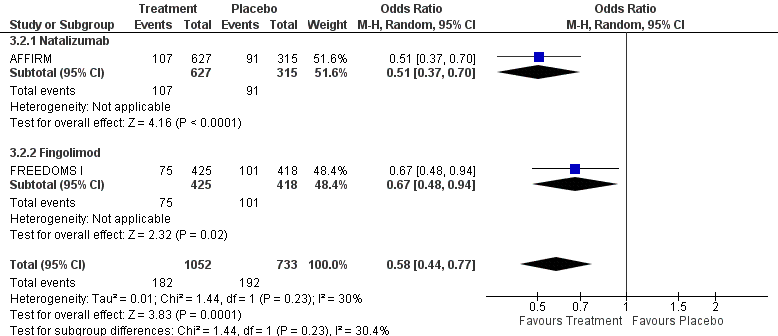


**Fig D.** Subgroup analysis on the annualized relapse rate reduction in observational studies of patients with relapsing remitting multiple sclerosis receiving treatment with Natalizumab of Fingolimod.


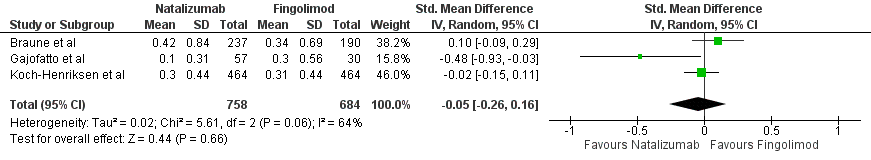


**Fig E.** Subgroup analysis on the annualized relapse rate odds ratios in observational studies of patients with relapsing remitting multiple sclerosis receiving treatment with Natalizumab of Fingolimod.


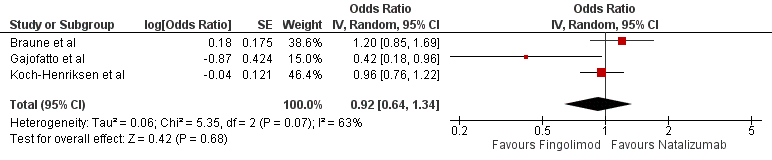


**Fig F.** Subgroup analysis on the proportion of relapsing remitting multiple sclerosis patients with disability progression receiving treatment with Natalizumab of Fingolimod in observational study protocols at both the first and second year.


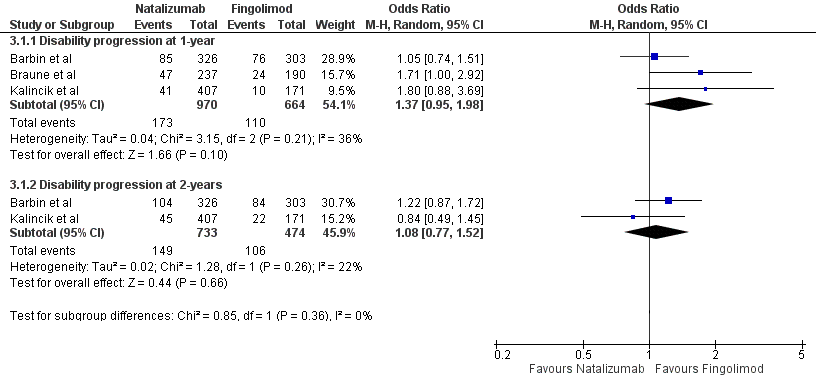


**Fig G.** Subgroup analysis on the proportion of relapsing remitting multiple sclerosis patients with no relapses receiving treatment with Natalizumab of Fingolimod in observational study protocols at both the first and second year.


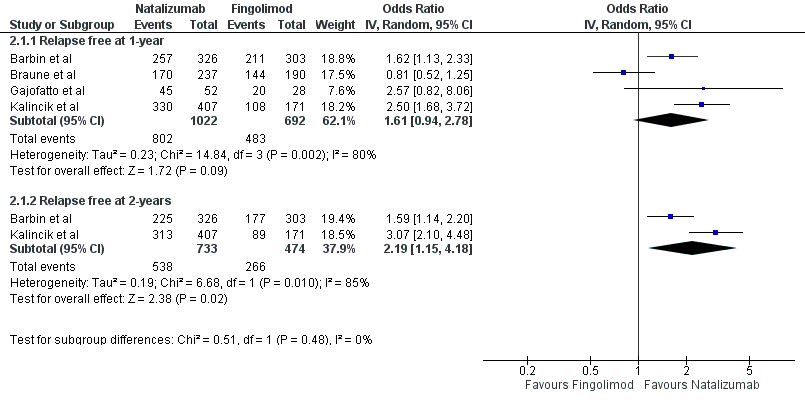


**Supplemental References**

1. Carruthers RL, Rotstein DL, Healy BC, Chitnis T, Weiner HL, Buckle GJ. An observational comparison of natalizumab vs. fingolimod using JCV serology to determine therapy. Mult Scler. 2014 Sep;20(10):1381-90

2. Cohen JA, Barkhof F, Comi G, Hartung HP, Khatri BO, Montalban X, et al. Oral fingolimod or intramuscular interferon for relapsing multiple sclerosis. N Engl J Med. 2010;362:402-15.

3. Kappos L, Antel J, Comi G, Montalban X, O'Connor P, Polman CH, et al. Oral fingolimod (FTY720) for relapsing multiple sclerosis. N Engl J Med. 2006;355:1124-40.

4. Kappos L, O'Connor P, Radue EW, Polman C, Hohlfeld R, Selmaj K, et al. Long-term effects of fingolimod in multiple sclerosis: the randomized FREEDOMS extension trial. Neurology. 2015;84:1582-91.

5. Miller DH, Khan OA, Sheremata WA, Blumhardt LD, Rice GP, Libonati MA, et al. A controlled trial of natalizumab for relapsing multiple sclerosis. N Engl J Med. 2003;348:15-23.

6. Rudick RA, Stuart WH, Calabresi PA, Confavreux C, Galetta SL, Radue EW, et al. Natalizumab plus interferon beta-1a for relapsing multiple sclerosis. N Engl J Med. 2006;354:911-23.

7. Barbin L, Rousseau C, Jousset N, Casey R, Debouverie M, Vukusic S, et al. Comparative efficacy of fingolimod vs natalizumab: A French multicenter observational study. Neurology. 2016;86:771-8.

8. Braune S, Lang M, Bergmann A; NTC Study Group. Second line use of Fingolimod is as effective as Natalizumab in a German out-patient RRMS-cohort. J Neurol. 2013;260:2981-5.

9. Gajofatto A, Bianchi MR, Deotto L, Benedetti MD. Are natalizumab and fingolimod analogous second-line options for the treatment of relapsing-remitting multiple sclerosis? A clinical practice observational study. Eur Neurol. 2014;72:173-80.

10. Kalincik T, Horakova D, Spelman T, Jokubaitis V, Trojano M, Lugaresi A, et al. Switch to natalizumab versus fingolimod in active relapsing-remitting multiple sclerosis. Ann Neurol. 2015;77:425-35.

11. Koch-Henriksen N, Magyari M, Sellebjerg F, Soelberg Sørensen P. A comparison of multiple sclerosis clinical disease activity between patients treated with natalizumab and fingolimod. Mult Scler. 2016 Apr 7. pii: 1352458516643393. [Epub ahead of print]
